# The complete chloroplast genome and phylogenetic analysis of *Smilax moranensis* (Liliales: Smilacaceae)

**DOI:** 10.1080/23802359.2022.2091960

**Published:** 2022-07-04

**Authors:** Baoyu Ji, Lixin Pei, Ning Cui

**Affiliations:** a School of Pharmaceutical Science and Technology, Tianjin University, Tianjin, China; b Central Laboratory, Shandong Academy of Chinese Medicine, Ji’nan, China; c College of Pharmacy, Henan University of Chinese Medicine, Zhengzhou, China

**Keywords:** *Smilax moranensis*, chloroplast genome, phylogeny

## Abstract

*Smilax moranensis* M.Martens & Galeotti 1842 is an important medicinal plant widely distributed in warm and temperate climates. In this paper, the complete chloroplast (cp) genome of *S. moranensis* was sequenced using the Illumina platform and assembled for the first time. This plastome is a circular structure of 157,907 bp in length. The GC content of the plastome was 37.16%. A total of 112 unique genes in this genome have been annotated, including 78 protein-coding genes, 30 transfer RNA genes, and four ribosomal RNA genes. Phylogenetic analysis based on complete cp genome sequences of Smilacaceae family showed that *Smilax* is monophyletic. The position of *S. moranensis* was positioned as the sister to the other seven *Smilax* species. These results provide an important basis for future species identification and taxonomic determinations, as well as the phylogenetic reconstruction of the family Smilacaceae.

The genus *Smilax* (Smilacaceae) comprises about 370 species, which are predominantly distributed in the tropical and temperate zones throughout the world, especially East Asia and North America (Plants of the World Online 2022). *Smilax moranensis* M.Martens & Galeotti 1842 is a woody vine with stems covered in spikes and is widely distributed in warm and temperate climates between 600 and 2000 m above sea level (Rosas-Ramirez et al. 2020). Several reports about the root of *S. moranensis* detail its pharmacological activities for treating rheumatic joint pain (Xu et al. 2014; Shu et al. 2017; Rosas-Ramirez et al. 2020). Despite the fact that *S. moranensis* has significant medicinal value, its research in genetics and evolution is still extremely rare. In this study, we reported the chloroplast (cp) genome of *S. moranensis* and examined its phylogenetic position within the family Smilacaceae. It is expected to lay the foundation for further molecular study and utilization of *S. moranensis*.

Fresh leaves of *S. moranensis* were collected from Xixia County, Nanyang City, Henan Province (33°38′N, 111°41′E). No permission was necessary for the *S. moranensis* collection, which is widely distributed in North China and is not listed as a national key protected plant. The specimen and DNA were deposited at the herbarium of Henan University of Traditional Chinese Medicine, Henan, China (contact person: Lixin Pei, xlpxlp@aliyun.com), under the voucher number HNPS2020-12-059. Total genomic DNA was extracted by using a Dneasy Plant MiniKit (Qiagen, Valencia, CA) according to the manufacturer’s instructions. Guided by our previous research basis (Cui et al. 2020), the DNA sample pretreatment, whole genome sequencing, cp genome assembly, junction validation, and cp genome annotation were performed in turn. The cp genome assembled and annotated here was submitted to the NCBI database (www.ncbi.nlm.nih.gov) with the GenBank accession number of OL693684.

The cp genome of *S. moranensis* was circular double-stranded DNA and displayed a typical quadripartite structure, including a pair of inverted repeat (IR) regions with lengths of 27,157 bp, separated by a large single-copy (LSC) region of 85,039 bp and a small single-copy (SSC) region of 18,554 bp. The GC content of the plastome was 37.16%. The genome harbored a set of 133 genes, of which 112 were unique genes. Totally, 85 protein-coding genes (78 unique genes) were annotated, which were mainly involved in processes related to photosynthesis and gene expression. Eleven protein-coding genes (*rps16*, *atpF*, *rpoC1*, *petB*, *petD*, *rpl16*, *rpl2*, *ndhA*, *ndhB*, *ndhB*, and *rpl2*) and eight tRNA genes (*trnK-UUU*, *trnG-UCC*, *trnL-UAA*, *trnV-UAC*, *trnI-GAU*, *trnA-UGC*, *trnA-UGC*, and *trnI-GAU*) contained one intron, and two genes (*ycf3* and *clpP*) contained two introns.

In order to reveal the evolutionary relationship of *S. moranensis*, the cp genomes of nine *Smilax* species were downloaded from the NCBI GenBank database. We aligned the plastomes using MAFFT and constructed a maximum-likelihood (ML) tree (Figure 1) by using RAxML 8.2.9 under the GTRGAMMA model with 1000 rapid bootstrap replicates (Cui et al. 2020). The ML tree indicated that the *Smilax* genus is monophyletic. *S. moranensis* was sister to the clade consisting of *S. microphylla* (NC056390), *S. nipponica* (NC049024), *S. riparia* (NC062359), *S. china* (NC049022 and HM536959), *S.* sp. (MW890012), *S. glycophylla* (NC049023), and *S. glabra* (NC058534 and MZ442610) with a bootstrap support value of 100%. The cp genome of *S. moranensis* will provide a theoretical basis to further understand the evolution of the family Smilacaceae and improve our understanding of its taxonomic classification.

**Figure 1.**
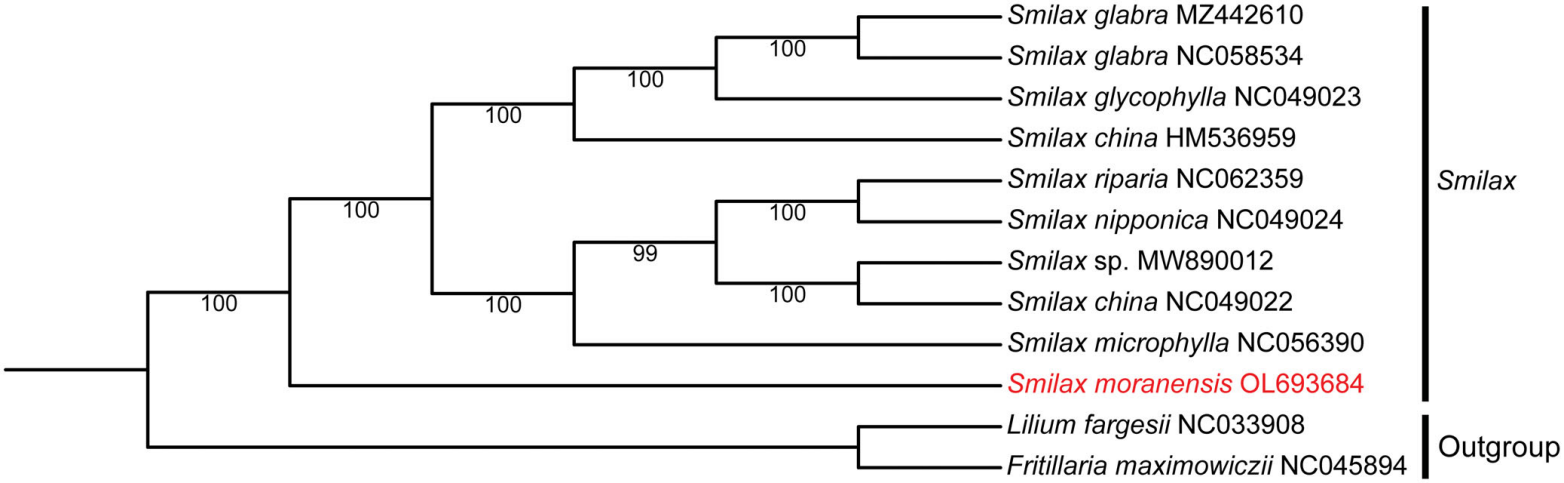
Phylogenetic tree constructed by using the whole chloroplast genome sequences of *Smilax* species with the maximum-likelihood method. Numbers near each branch are the bootstrap values.

## Author contributions

N. Cui designed and conceived this work; L.X. Pei collected the samples and carried out the experiment; B.Y. Ji analyzed the data and wrote the first version of the manuscript. All authors read, revised, and approved the final manuscript.

## Ethical approval

No permission was necessary in this study for the sample collection. *Smilax moranensis* is widely distributed in North China and is not listed as a national key protected plant.

## Data Availability

The complete chloroplast genome sequence of *Smilax moranensis* was deposited in the GenBank database (https://www.ncbi.nlm.nih.gov/genbank, OL693684). Raw sequencing reads used in this study have been deposited in the SRA database (https://www.ncbi.nlm.nih.gov/sra). The associated BioProject, SRA, and Bio-Sample numbers are PRJNA785329, SRR17085809, and SAMN23553129, respectively.
